# Expression of Cellular Components in Granulomatous Inflammatory Response in *Piaractus mesopotamicus* Model

**DOI:** 10.1371/journal.pone.0121625

**Published:** 2015-03-26

**Authors:** Wilson Gómez Manrique, Gustavo da Silva Claudiano, Marcello Pardi de Castro, Thalita Regina Petrillo, Mayra Araguaia Pereira Figueiredo, Marco Antonio de Andrade Belo, María Isabel Quiroga Berdeal, Julieta Engracia Rodini de Moraes, Flávio Ruas de Moraes

**Affiliations:** 1 Department of Veterinary Pathology, School of Agrarian and Veterinary Sciences, Sao Paulo State University, Jaboticabal campus, São Paulo, Brazil; 2 Department of Pathological Anatomy, College of Veterinary, University of Santiago de Compostela, Lugo, Spain; Fundació Institut d’Investigació en Ciències de la Salut Germans Trias i Pujol. Universitat Autònoma de Barcelona. CIBERES, SPAIN

## Abstract

The present study aimed to describe and characterize the cellular components during the evolution of chronic granulomatous inflammation in the teleost fish pacus (*P*. *mesopotamicus*) induced by Bacillus Calmette-Guerin (BCG), using S-100, iNOS and cytokeratin antibodies. 50 fish (120±5.0 g) were anesthetized and 45 inoculated with 20 μL (40 mg/mL) (2.0 x 10^6^ CFU/mg) and five inoculated with saline (0,65%) into muscle tissue in the laterodorsal region. To evaluate the inflammatory process, nine fish inoculated with BCG and one control were sampled in five periods: 3^rd^, 7^th^, 14^th^, 21^st^ and 33^rd^ days post-inoculation (DPI). Immunohistochemical examination showed that the marking with anti-S-100 protein and anti-iNOS antibodies was weak, with a diffuse pattern, between the third and seventh DPI. From the 14^th^ to the 33^rd^ day, the marking became stronger and marked the cytoplasm of the macrophages. Positivity for cytokeratin was initially observed in the 14^th^ DPI, and the stronger immunostaining in the 33^rd^ day, period in which the epithelioid cells were more evident and the granuloma was fully formed. Also after the 14^th^ day, a certain degree of cellular organization was observed, due to the arrangement of the macrophages around the inoculated material, with little evidence of edema. The arrangement of the macrophages around the inoculum, the fibroblasts, the lymphocytes and, in most cases, the presence of melanomacrophages formed the granuloma and kept the inoculum isolated in the 33^rd^ DPI. The present study suggested that the granulomatous experimental model using teleost fish *P*. *mesopotamicus* presented a similar response to those observed in mammals, confirming its importance for studies of chronic inflammatory reaction.

## Introduction

The inflammatory response of fish is generally similar to that of mammals. However, improvements in the human tuberculosis pathogenesis have been obtained using teleost fish model [1]. The acute response is exudative, with production of fluid, proteins and cells [2–3, 4]. The chronic response is proliferative and its main components are mononuclear cells, connective tissue and neoformed vessels [5–6].

When the causal agent of the acute inflammation persists, it becomes chronic and gives rise to granuloma. This can occur both in immune and in non-immune forms, which differ in relation to the characteristics of the macrophages accumulated: whether the activity is strictly phagocytic or as secretory epithelioid cells [7] and in some cases, with formation of giant multinucleated cells, in accordance with the phylogenetic evolution of the fish species considered [8–9].

Immunolabeling of macrophages and epithelioid cells in granulomas induced by Bacillus Calmette-Guerin (BCG) in *Arius genus* led to understanding of how granulomas formed in several fish species in different positions on the phylogenetic scale [10]. Use of immunolabeling with poly and monoclonal antibodies in fish infected with *M*. *paratuberculosis* and *M*. *avium* demonstrated that immunohistochemistry is valid for diagnosing mycobacteriosis in fish [11], and also valid for *M*. *marinum* [12].

On the other hand, identification of nitric oxide (NO) as a molecule mediating the inflammatory response [13] has encouraged researches for identifying the presence and participation of this and its derivatives in this event. Thus, through using PCR, the expression of the messenger RNA sequence that codes for inducible nitric oxide synthase (iNOS) has been demonstrated, thus showing its participation in chronic inflammation in fish, in a similar way to what has been observed in mammals [14].

Study conducted on *O*. *mikyss* has suggested that iNOS participates in the immune response with a protective function against pathogenic agents [15]. The S-100 family of calcium-binding proteins is present in neutrophils and monocytes, and it has been correlated with maintenance of the granuloma [7], but has not been identified in fish.

Other studies were performed with pacus to study the chronic inflammatory response [6–16]. The species chosen is a native teleost fish of the Parana-Paraguay Basin, and is of importance in Brazil for human consumption, angling and aquaculture. The pacu has proven to be a good bioindicator of water quality, and in accordance with Castro et al. [17] this species has been used in ecotoxicity studies for registration of chemicals in Brazil. The present study aimed to describe and characterize the cellular components during the evolution of chronic granulomatous inflammation in pacus (*Piaractus mesopotamicus*) induced by Bacillus Calmette-Guerin, using S-100, iNOS and cytokeratin antibodies.

## Material and Methods

### Fish and procedures

Fifty male or female of *P*. *mesopotamicus* (120±5.0 g) were randomly distributed into six tanks (250 L). After conditioning for seven days, the fish were anesthetized in an alcoholic solution of benzocaine (0.1 g/mL) (1:1,0000 anesthesia/water) to minimize suffering, and 45 fish were inoculated with 20 μL (40 mg/mL) (number of live bacilli greater than 2.0 x 10^6^ CFU/mg of the Mureau BCG strain, Rio de Janeiro) and five animals were inoculated with saline solution 0,65% (control). The inoculation was done into muscle tissue in the laterodorsal region, equidistant between the start of the dorsal fin and the midline. The fish were returned to their tanks, with continuous water flow (outflow of 1.0 L/min) and constant aeration. They were fed with commercial feed (3% of the biomass, 28% of GP and 4000 kcal of GE kg^-1^). The quality of the water remained within the adequate range for fish comfort [18], (dissolved oxygen = 5.7 ± 0.5 mg/L; temperature = 25.9 ± 1.3°C; potential of hydrogen ions (pH) = 7.3 ± 0.5; and electric conductivity = 117.9 ± 8.6 μS/cm), probe using YSI Model MPS 556.

All procedures were carried out in accordance with the Guide for the Care and Use of Laboratory Animals and the experimental protocol was approved by the Committee of Ethics in the use of Animals, CEUA—“Comissão de Ética no uso de Animais” (protocol n° 020092/09) from the São Paulo State University.

To evaluate the inflammatory process, five subgroups of nine fish inoculated with BCG and one control fish inoculated with saline solution (0,65%) were harvested from the inoculated group, on the 3^rd^, 7^th^, 14^th^, 21^st^ and 33^rd^ days post-inoculation (DPI). The euthanasia was realized by fish immersion in an alcoholic solution of benzocaine (0.1 g/mL) (500 v/v anesthesia/water), fragments of the inoculated muscle were collected, fixed in Bouin solution for three hours and transferred to 10% formol solution. These samples then underwent routine histological processing in order to prepare slides for histopathological and immunohistochemical analysis.

### Histopathology

Paraffin-block sections of thickness 5 μm were cut and mounted on slides. These were stained with hematoxylin-eosin (HE) for histopathological examination and with Ziehl-Neelsen (ZN) reagent to identify the bacillus. The mounted slides were examined under a light microscope (Olympus BX 51) and were photographed (OlympusDP72 and CellSens software v. 1.5).

### Immunohistochemistry

Sections of thickness 3 μm were cut and mounted on slides that were surfaced with poly-L-lysine for better adhesion. The slides were then deparaffinized and blockage of endogenous peroxidase activity was then performed (Dako S2023) for 30 min, followed by antigen recovery, done in a pressure pan with a buffer solution of 10 mM sodium citrate, at pH 6.0, for 15 min. For immunolabeling of cytokeratins, WSS antibodies (Wide Spectrum Screening, Dako N1512) were used at a dilution of 1:500 v/v. For macrophages, anti-S-100 protein antibodies (Neomarkers RB-044-A) were used at a dilution of 1:200 v/v. For iNOS antibodies (Neomarkers RB-1605-P), the dilution was 1:3,000 v/v. From the latter, no antigen recovery was performed. The antibodies were incubated for two hours in a damp chamber at room temperature (23°C). The secondary substrate used was anti-rabbit polyclonal antibodies (Dako EnVision + Dual Link System—HRP—K4061). The samples were developed using the chromogen 3,3'-diaminobenzidine (DAB, Dako K4010–4011) (1:200 v/v).

For the positive control, fish skin was used for cytokeratin, human nervous system for S-100 and fish kidney for iNOS. For the negative control, it was decided to exclude the primary antibody while maintaining the other steps. Counterstaining was done using Harris hematoxylin and mounting was done using DePex (Gurr).

Digital images for five photomicroscopy of each animal (nine animals per period of analysis, n = 45) were captured using a digital camera (Olympus DP 72, with Cell Sens v.1.5 imaging software). A blinded experienced pathologist performed immunohistochemistry analyses and the histopathological findings related to intensity of immunostaining were scored varying from 0 (without immunostaining), +1 (Weak = 10 to 20% of positive cells), +2 (Moderate = 20 to 50%) and +3 (Strong = ≥50%) [19–20].

### Statistical analysis

For statistical analyses, data from immunostaining (scored 0 to 3) was presented as mean values (n = 45). Comparison of the different experimental days for each antibodie was carried out by applying a PROC ANOVA procedure, using the Statistical Analyses System [21], and normality of residuals was assessed for iNOS, S100 and Cytokeratin levels each analysed separately to assure valid analyses. Significant differences (P< 0.05) were estimated on the basis of T test, according to Snedecor and Cochran [22].

## Results

The histopathological examination using the Ziehl-Neelsen staining showed that acid and alcohol-fact bacilli were present in BCG inoculated fish and absent in controls. After the third day, there were extensive foci of mononuclear cellular infiltrate surrounding the necrotic centers, with dissociation of muscle fibers caused by edema only in BCG inoculated fish. Giant cells progressively appeared and went on increasing in size and number of nuclei with increasing duration of chronicity. Giant cells characterized as foreign-body type were present between the third and seventh days.

After the 14^th^ day, some giant cells of Langhans type were observed, characterized by arrangement of the nuclei on the periphery of the cytoplasm. Over the course of time, the inoculated region presented diminishing edema, areas of necrosis, and increasing quantities of macrophages in the inflammatory infiltrate, at the focus of the lesion. Also after the 14^th^ day, a certain degree of cellular organization was observed, due to the arrangement of the macrophages around the inoculated material, with little evidence of edema. At this time, melanomacrophages and cells with the characteristics of fibroblasts were seen. In the more external parts of the granuloma, mononuclear infiltrate was observed with scarce cytoplasm cells and a well-stained evident nucleus, and with condensed chromatin, which suggested that lymphocytes were more concentrated in the more external perivascular regions of the granuloma. The more advanced the chronicity of the inflammatory process was, the more evident the cellular organization was.

**Fig 1:**
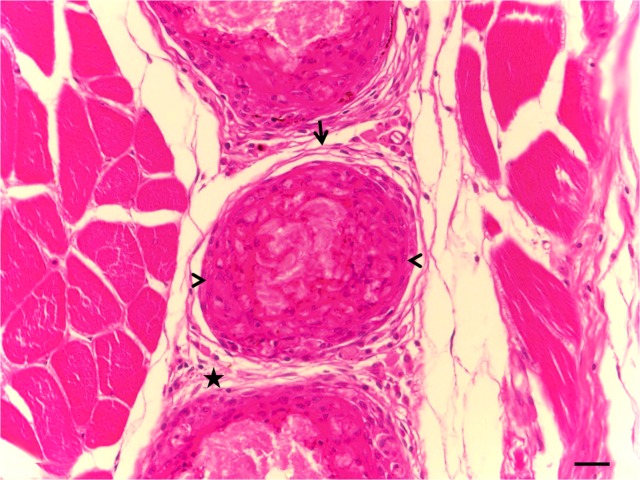
Photomicrograph of the muscle tissue of *Piaractus mesopotamicus*, 33 days after inoculation of BCG. Mononuclear infiltrate (star), fibroblasts arranged around the granuloma (arrow) and macrophages arranged around the inoculum (arrowhead) can be seen. (HE). Bar = 20 μm.

The arrangement of the macrophages around the inoculum, the fibroblasts, the lymphocytes and, in most cases, the presence of melanomacrophages formed the granuloma and kept the inoculum isolated after 33 days (Fig. 1).

**Fig 2:**
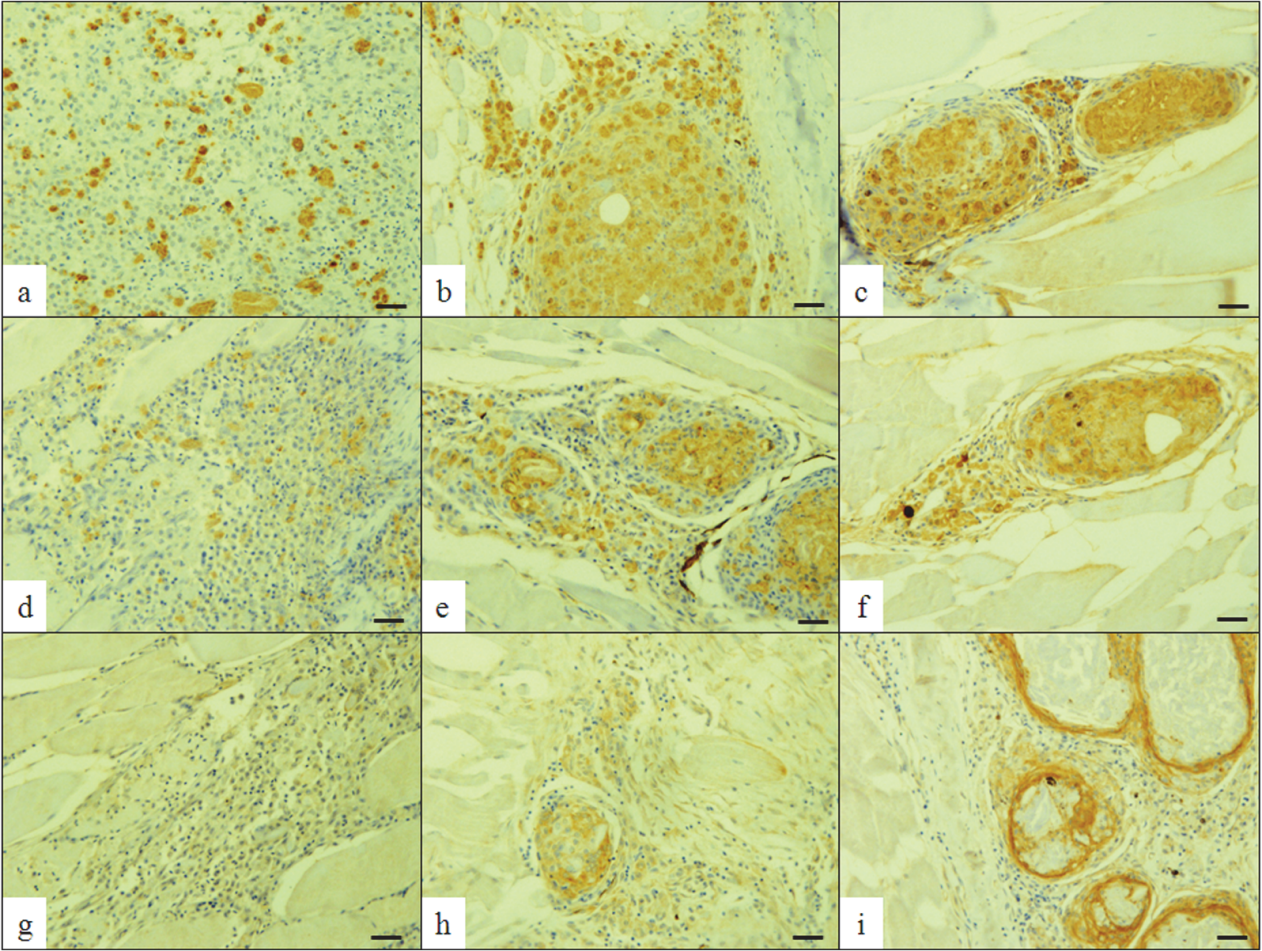
Photomicrograph of the muscle tissue of *Piaractus mesopotamicus*. Positive immunolabeling of macrophages for anti-S-100 protein, 3 (a), 14 (b) and 33 (c) days after inoculation of BCG; Immunolabeling of macrophages with anti-iNOS, 3 (d), 14 (e) and 33 (f) days after inoculation of BCG. Immunolabeling of epithelioid cells with anti-cytokeratin, 3-negative (g), 14 (h) and 33 (i) days after inoculation of BCG. DAB. Bar = 50 μm.

Only in fish inoculated with BCG, immunohistochemical examination showed that the marking with anti-iNOS antibodie was strong in the initial phase third and seventh days after inoculation, decreasing significantly (P<0.0001) from the 14th day (Fig. 2- A,B,C, and Table 1). Anti-S-100 protein antibodie was weak, with a diffuse pattern, between the third and seventh days after inoculation (Fig. 2 and Table 1). From the 14^th^ to the 33^rd^ day, the marking became stronger (P<0.0001) and marked the cytoplasm of the macrophages (Fig. 2 and Table 1), thus demonstrating that with increasing chronification, the marking became more evident and restricted to the granuloma. Positivity for cytokeratin was initially observed in the 14^th^ day after inoculation, and the stronger immunostaining in the 33^rd^ day, period in which the epithelioid cells were more evident and the granuloma was fully formed (Fig. 2 and Table 1).

**Table 1:** Mean values (n = 45)^1^ and ANOVA^2^ for score of iNOS, S100 and cytokeratin intensity of immunostaining^3^ observed in the muscle tissue of pacus inoculated with BCG.

Period	iNOS	S100	Cytokeratin
3	2.77^A^	1.25^D^	0.00^C^
7	2.81^A^	1.40^D^	0.00^C^
14	2.51^B^	1.84^C^	1.73^B^
21	2.46^B^	2.48^B^	2.33^AB^
33	1.80^C^	2.73^A^	2.86^A^
F value	20.63	43.20	38.05
Pr>F^4^	<.0001	<.0001	<.0001
CV^4^	28.53	26.93	80.27

^1^ Mean values (n = 45: nine fish X five fields analyzed per animal) with at least one letter in common do not differ by the T test.

^2^ The analysis of variance within each parameter was represented by capital letters (columns) for comparison of data from each treatment through the experimental days.

^3^ Immunostaining score: 0 (without immunostaining), +1 (Weak = 10 to 20% of positive cells), +2 (Moderate = 20 to 50%) and +3 (Strong = ≥50%).

^4^ Pr>F: Probability of significance associated to the F value/ CV: Coefficient of variation.

## Discussion

In the present study, the chronic inflammatory process induced by BCG gave rise to formation of immune-type granuloma with immunolabeling of macrophages and epithelioid cells, but not polykaryon giant cells, which are a particular characteristic of modern fish, as described by Sado and Matushima [10]. During the inflammatory event, a proliferative process was observed initially, and the start of phagocytic activity by the macrophages was identified through marking with S-100 antibodies, in a process similar to what has been seen in mammals [23]. This marking has mainly been correlated with cell differentiation [24], cell transduction and transcription [25] and the possible actions of some cytokines that have intra and extracellular activity similar to that of S-100 protein [26]. It is possible that in pacus this process is similar.

The immunolabeling with iNOS from the third DPI on wards makes it possible to infer that in pacus macrophages present receptors earlier on, or that chemical mediators such as interleukin 1 (IL-1) are released earlier and then marked. In other species, marking with iNOS [27] and interferon-gamma (IFNγ) [28] occur in this manner. This immunolabeling may also be related to the response generated by macrophages, with production of reactive oxygen species (ROS) and reactive nitrogen species (RNS) in *P*. *mesopotamicus*, as observed in mammals [15].

The evolution of the granuloma in *P*. *mesopotamicus* began with infiltration of macrophages and, over the course of time, these became organized and arranged around the inoculum, but without formation of a caseous center, unlike what was observed by Romano et al. [12] in flounders (*Paralichthys orbignyanus*) and barber goby (*Elacatinus fígaro*), in which *M*. *marinum* was diagnosed, i.e. an active species differing from what was used in the present study. The cell staining characteristics from the present observations in *P*. *mesopotamicus* demonstrated that the macrophages with phagocytic activity underwent morphophysiological modifications and became transformed into epithelioid cells with secretory activity, as also observed by Gauthier et al. [29] in striped bass (*Morone saxatilis*). This finding was confirmed through the immunolabeling with anti-cytokeratin, and these results were compatible with those obtained by Noga et al. [30] in *O*. *niloticus*, for characterizing the same event.

The understanding of the physiopathological issues involved in the genesis of lesions caused by different species of mycobacteria remains insufficient. Thus, use of new cellular markers may help in achieving better understanding of these processes and thus contribute towards the search for solutions, particularly from the point of view of comparative pathology.

According to the hypothesis of Howe et al. [31] mammals, including humans, retain significant genetic homology when compared to teleostean fishes. The present study suggested that the granulomatous experimental model using teleost fish *P*. *mesopotamicus* presented a similar response to those observed in mammals, confirming its importance for studies of chronic inflammatory reaction.
